# Interferon alpha-2b treatment for exophytic nasal papillomas and human papillomavirus infection^☆^

**DOI:** 10.1016/j.bjorl.2024.101449

**Published:** 2024-06-03

**Authors:** Popova Inga, Tregub Pavel, Degtyarevskaya Tatiana, Starostina Svetlana, Shadyev Timur, Apollonova Irina, Boyko Andrey, Petrovskii Vladimir, Kozlova Anastasia, Ibrahimli Irada

**Affiliations:** a I.M. Sechenov First Moscow State Medical University, Moscow, Russian Federation; b Clinic Central LLC, Moscow, Russian Federation; c RUDN University, Moscow, Russian Federation; d Brain Science Institute, Research Center of Neurology, Moscow, Russian Federation; e Bauman Moscow State Technical University, Moscow, Russian Federation

**Keywords:** Exophytic sinonasal papilloma, Recombinant human interferon alpha-2b, Human Papillomavirus

## Abstract

**Objectives:**

Exophytic Sinonasal Papilloma (ESP) is a benign tumor of the sinonasal tract. Complete surgical excision by endoscopic surgery is the treatment of choice. However, a high recurrence rate (36% at 5-year follow-up) is associated with this method, which may indicate the presence of microorganisms such as Human Papillomavirus (HPV). It is important to note that the standard treatment for ESP does not include antiviral drugs. In our study, we are testing the effectiveness of an interferon-containing drug in reducing recurrence and postoperative reactions in patients with ESP.

**Methods:**

We included 78 patients aged 23–83 years with a confirmed diagnosis of ESP by rhinoscopy and nasal endoscopy and a positive PCR test for HPV in nasal scrapings. To compare the results, we divided the patients into main and control groups. The main group received recombinant human interferon after surgery, while the control group did not receive the drug. We performed a statistical analysis to compare the proportion of patients without reactive manifestations at different stages of the postoperative period, as well as to compare the proportion of patients with recurrent ESP at certain stages of observation.

**Results:**

The introduction of recombinant human interferon accelerated the resolution of postoperative reactions and promoted the healing of the nasal mucosa after surgical removal of the ESP. We also found a statistically significant association between treatment with recombinant interferon and a reduction in the recurrence rate of ESP.

**Conclusion:**

According to the results of the study, it was found that in the main group of patients who received rhIFN-α2b (recombinant human Interferon alpha 2b) in the postoperative period, the frequency of relapses of ESP and the time of postoperative recovery were significantly lower than in patients in the control group who did not take the drug.

**Level of evidence:**

Cohort Study.

## Highlights

• ESP treatment with rhIFN-α2b is effective due to elimination of HPV.
• The introduction of rhIFN-α2b accelerated the resolution of postoperative reactions.
• The introduction of rhIFN-α2b promoted the healing of the nasal mucosa after surgical removal of the ESP.

## Introduction

Exophytic Sinonasal Papilloma (ESP) is a benign tumor of the sinonasal tract that arises from sinonasal (Schneiderian) mucosa ‒ an ectodermally derived epithelium comprising respiratory-type pseudostratified ciliated columnar epithelial cells, inconspicuous basal cells, and various mixed mucus-producing goblet cells. It is the second most common type of sinonasal papillomas, with an incidence of 0.72–2.3 cases per 100,000 population per year.^1^ ESP is most common in young and middle age, with a male to female ratio of 10:1. ESP usually occurs in the region of the nasal septum, also in the nasal vestibule or middle turbinate area. In majority of patients, the process is unilateral; bilateral lesion is rare. Clinical manifestations of ESP include nasal obstruction, nasal discharge or epistaxis, postnasal drip, headache, hyposmia, and rhinorrhoea, which significantly compromise the quality of life.^2^, ^3^

Complete surgical excision through endoscopic surgery is the treatment of choice for ESP. However, this method is associated with a high recurrence rate of the neoplasm, reaching 36% during a 5-year follow-up.^4^, ^5^ It has been suggested that the high recurrence rate of exophytic nasal papillomas can be attributed to microorganisms found in a pathological hearth, such as Human Papillomavirus (HPV).^6^, ^7^, ^8^ HPV has a number of features that promote the development of inflammatory processes and growth of neoplasms on the mucous membrane of the upper respiratory tract.^9^, ^10^ Taking into consideration that the nasal mucosa, from an anatomical and physiological points of view, represents the surface through which the host interacts with the environment, the recurrences may be caused by re-infections, or they may be explained by failure of standard surgical treatment to eliminate the HPV. It is important to note that standard treatment for ESP does not include antiviral drugs. Therefore, in the present study, we suggested that administration of an interferon-containing drug can mitigate the viral infection in the nasal mucosa and thereby facilitate the resolution of postoperative reactive phenomena and reduce the rate of ESP recurrence. To verify this hypothesis, we compared these outcomes in patients with ESP and a positive HPV DNA (Deoxyribonucleic Acid) test who underwent standard surgical treatment and those with combined surgical and topical recombinant human Interferon alpha-2b (rhIFN-α2b) treatment.

## Methods

The study was conducted in accordance with the Helsinki Declaration. All patients provided written informed consent prior to being included in the study. All studies were conducted according to international rules for handling human biomaterial and approved by the local ethical committee of the I.M. Sechenov First Moscow State Medical University, Moscow, Russian Federation (Protocol nº 11–23). The examination of patients was carried out in the Clinic Central LLC, Moscow, Russian Federation.

### Patients and study design

A total of 78 patients with ESP (age range, 23–83 years) were prospectively enrolled in this study between June 2019 and December 2021. ESP was diagnosed based on the clinical (rhinoscopy, nasal endoscopy) and pathomorphological examination. The inclusion criteria were diagnosis of ESP of the nasal vestibule and positive HPV DNA test for the presence of HPV types with high or low oncogenic risk in the lesion. In all patients, the papilloma had the same degree of manifestation (the extent of the disease): it was located in the vestibule of the nose on the nasal septum or on the lateral wall of the nasal cavity with a relatively wide base, and had dimensions ranging from 2 to 7 mm. The non inclusion criteria were: patients younger than 18 or older than 85 years; presence of severe somatic diseases; patients with contraindications to the use of therapy used in the study; pregnancy; patients under the influence of psychotropic drugs; the inability and unwillingness of the patient to give voluntary informed consent to participate in the study or to fulfill the requirements of the study.

Criteria for excluding patients from the study: refusal of the patient to observe upon detection or exclusion of the patient from the research physician.

The included patients were randomized into 2 groups: Group A included 38 patients who underwent only surgical treatment (ESP removal) (control group), Group B included 40 patients who underwent surgical treatment and in the postoperative period local treatment with rhIFN-a2b (main group).

Surgical treatment for all patients was performed by a team consisting of 2 doctors and 1 nurse who were not informed about the patient's affiliation with a specific group. Postoperative monitoring was carried out by one doctor who also did not have information about the patient's assigned group.

### Surgical treatment

All surgeries were performed as day-care procedures. The nasal mucosa was anesthetized by infiltration with 1% lidocaine. The tumor excision was performed using the 20 W VersaPulse PowerSuite Laser (Lumenis, Israel). The laser was set at a pulse energy of 0.6 J with a frequency of 12 Hz. During vaporization, the laser beam was tangent to the surface. After the removal of ESP, the excision area was treated with saline solution. Bleeding was not observed, and patients did not require anterior tamponade. All removed tissue was sent for pathomorphological examination.

### Postoperative treatment and follow-up

In both groups, postoperative care included irrigation of the nasal cavity with warm sterile normal saline by a patient 3 times/day for 14 days. Additionally, Group B patients were administered rhIFN-α2b nasal drops (3 drops/nostril/time, 4 times/day) for 4 weeks. The severity of postoperative reactive phenomena was assessed on days 1, 7, 14, and 21 after surgery by evaluating tissue edema, hyperaemia in the area of ESP excision, and vascular pattern in the postoperative area and graded on a scale from 0 to 3: 0 – No reactive phenomena, 1 – Weakly expressed phenomena, 2 – Moderately pronounced phenomena, 3 – Very pronounced reactive phenomena.

The ESP recurrence and the presence of HPV DNA in the nasal mucosa were evaluated at 3- and 6-month follow-up visits.

### Identification of HPV by real-time PCR

Identification of HPV DNA in scrapings from the nasal mucosa was performed by means of the RealBest DNA HPV HR (Human Papillomavirus High-Risk) genotype kit (targets HPV types with high oncogenic risk: types 16, 18, 31, 33, 35, 39, 45, 51, 52, 56, 58, and 59) and RealBest DNA HPV 6/11/44 kit (targets HPV types with low oncogenic risk: types 6, 11, and 44) (Vector-Best, Novosibirsk, Russia). All amplification procedures were performed using a DTprime (DNA-Technology prime) detecting thermocycler (DNA-Technology, Moscow, Russia) according to the manufacturer’s instructions.

### Statistical analysis

Statistical analysis was performed with IBM (International Business Machines Corporation) SPSS Statistics (Statistical Package for the Social Sciences) (version 23.0; IBM Corp.). Continuous variables were presented as median, range, and/or Interquartile Range (IQR); categorical variables were presented as number, share and/or percentage. The Mann-Whitney *U* test and Chi-Square test were used to compare the patient groups for baseline demographic and clinical characteristics, respectively. The Fisher’s exact test with the Šidák-Holm adjustment was used to compare the proportions of patients without reactive manifestations on postoperative days 1, 7, 14, and 21 as well as the proportions of patients with ESP recurrence at 3- and 6-month follow-up in Group A vs. Group B. The McNemar’s test was used to compare the proportions of patients with ESP recurrence in the groups at 3 vs. 6 month follow-up. To evaluate the effect of the complementary treatment with rhIFN-α2b on the rate of resolution of postoperative reactive phenomena taking into consideration that the same patients were examined at four postoperative time points, a Generalized Estimating Equation (GEE) was used, with presence of reactive phenomena (“yes” or “no”) as a binary outcome, the type of treatment (“surgery” or “surgery + rhIFN-α2b”) as a categorical predictor variable, time (postoperative days 1, 7, 14, and 21) as a continuous predictor variable, and interaction term of the two predictor variables. To investigate the relationship between ESP recurrence at 6-month follow-up as a binary outcome and age, sex, HPV oncogenic risk, and type of treatment as predictor variables, a binomial logistic regression was used. Two-tailed p-values of less than 0.05 were considered statistically significant.

## Results

A total of 78 patients with ESP (35 men and 43 women) were included in the study. The median age was 42 years (range: 23–83 years, IQR: 36–55 years). The most frequent complaints were discomfort in the nasal vestibule (94.8%), itching (67.9%), sensation of a foreign body on palpation (74.3%), and difficulty in nasal breathing on the side of ESP formation (80.7%). All ESPs were located on either the side wall of the nasal cavity or the nasal septum. Infection with high- or low-risk HPV was detected in 54 (69.2%) and 24 (30.8%) patients, respectively. The sizes of papillomas were from 0.5 to 3.5 mm, papillomas were on a narrow base. Anamnestic data indicated the primacy of the process, the duration of HPV persistence was not previously determined. The patients were randomly assigned to either Group A (surgical treatment only) or Group B (surgical treatment supplemented with topical rhINF-α2b). No significant difference in patients’ age, male/female ratio, HPV oncogenic risk, and ESP localization was found between the groups (Table 1).

**Table 1:** Patient’s demographic and clinical characteristics.

Characteristic	Surgery (n = 38)	Surgery + IFN (n = 40)	*p*-value
Age (years)	43 (36–54)	41 (35–56)	0.825
Sex (n)			0.816
Male	18	20	
Female	20	20	
HPV type (n)			0.734
High oncogenic risk	27	27	
Low oncogenic risk	11	13	
Localization (n)			0.622
Nasal septum	15	18	
Lateral wall of the nose	23	22	

### Postoperative reactive phenomena

**Figure 1:**
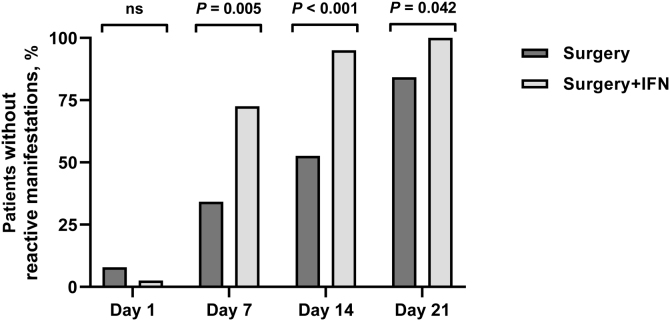
Reactive changes in groups A and B on days 1, 7, 14 and 21 after surgery.

Local signs of postoperative reactive phenomena were evaluated on days 1, 7, 14, and 21 after surgery. As shown in Fig. 1, on day 1, the percentage of patients without visible signs of inflammation was low in both groups (*p* = 0.824); but on days 7, 14, and 21, it was significantly higher in Group B (surgery + rhIFN-α2b) compared to Group A (surgery) (*p* = 0.005, *p* < 0.001, and *p* = 0.042, respectively).

**Figure 2:**
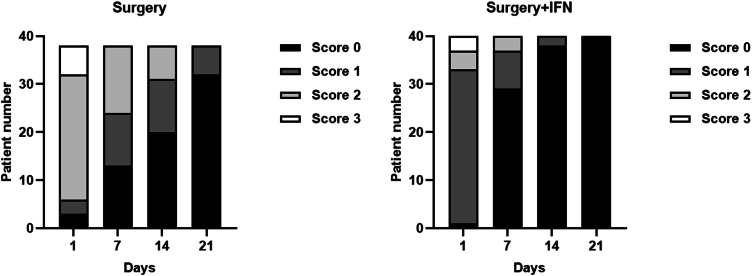
The severity of reactive phenomena and their regression in the group of patients with surgical treatment and in the group with surgical treatment and the use of interferon.

The severity of reactive phenomena in groups A and B is shown in Fig. 2. Severity is measured in scores from 0 to 3 where score 0 corresponds to no reactive phenomena and score 3 corresponds to very pronounced reactive phenomena. On the 1st day after operation patients with all available scores are present in Group A (Surgery) and Group B (Surgery + IFN). On the 7th day quantity of patients with score 0 is more in Group B than in Group A. And on the 21st day all patients in Group B have score 0 of reactive phenomena.

**Figure 3:**
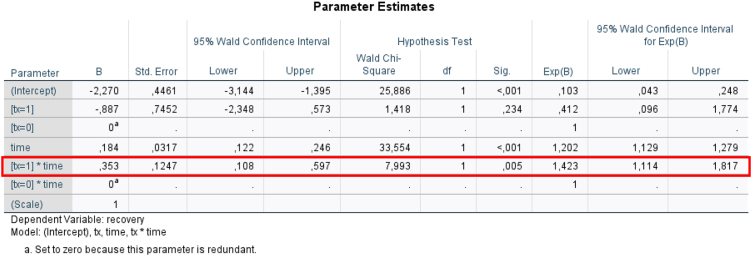
Parameters estimates for the general estimating equation.

To further evaluate the effect of rhIFN-α2b administration on resolution of inflammation and healing process in the postoperative period, we performed a GEE analysis. As shown in Fig. 3, the treatment × time interaction was statistically significant (*p* = 0.005), suggesting a greater rate of resolution of postoperative reactive phenomena in Group B than in Group A. Collectively, our data demonstrate that administration of rhIFN-α2b in the postoperative period promotes healing and repair processes in the nasal mucosa after surgical excision of ESP.

### ESP recurrence at 3- and 6-month follow-ups

**Figure 4:**
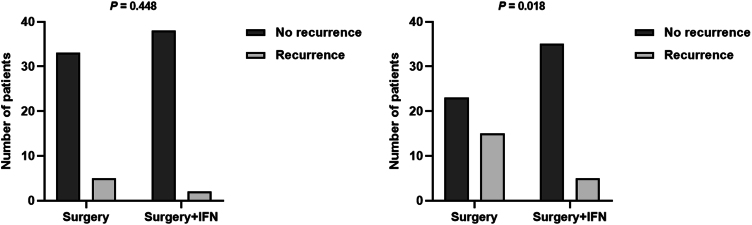
Assessment of relapses after 3 and 6 months of follow-up.

At a 3-month follow-up, the frequency of recurrence of ESP in groups A and B was the same (13.2% and 5.0%, respectively, *p* = 0.448); but at a 6-month follow-up, it was significantly higher in Group A than in Group B (39.5% vs. 12.5%, respectively, *p* = 0.018) (Fig. 4). Moreover, the increase in the percentage of patients with recurrent ESP was significant in Group A (from 13.2% to 39.5%, *p* = 0.002) but not in Group B (from 5.0% to 12.5%, *p* = 0.250). No significant difference was found in the proportion of high-risk HPV and male sex in patients with recurrent ESP compared with patients without recurrent ESP in either Group A or Group B (data not shown).

**Figure 5:**
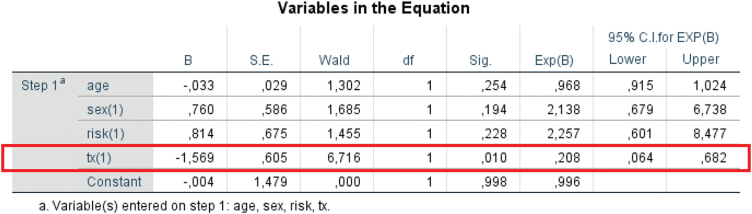
Variables in the binomial logistic regression equation.

To further investigate the relationships between patients’ clinical characteristics and ESP recurrence at a 6-month follow-up, we performed a binomial logistic regression with age, sex, HPV oncogenic risk, and a type of treatment as predictor variables and with presence or absence of ESP recurrence as a binary outcome. As shown in Fig. 5, only a type of treatment was statistically significantly associated with the outcome. Specifically, the treatment with rhIFN-α2b decreased the odds of ESP recurrence by a factor of 4.76 (i.e., 1/0.21) (*p* = 0.010).

## Discussion

HPV is one of the underestimated etiological factors of ESP accounting for up to 100% of all ESP cases.^4^ However, the standard treatment for ESP includes only surgical excision of the tumour,^11^, ^12^, ^13^ which cannot guarantee eradication of HPV and, therefore, may be a cause of high ESP recurrence rate, reaching 60%.^14^ In this regard, we hypothesized that ESP patients could benefit from supplementation of surgical tumour excision with topical antiviral treatment. To test this hypothesis, we enrolled 78 patients with ESP and detectable HPV DNA on the tumor surface. About two-thirds of them were diagnosed with high-risk HPV and one-third with low-risk HPV. According to Glâtre et al., the prevalence of HPV in individuals with ESP is 100%. At the same time, the growth of neoplasms is mainly caused by HPV of low oncogenic risk, such as types 6 and 11.^4^, ^5^, ^6^, ^7^ This can be explained by the sample of a specific study.

The recovery time after removal of HPV-associated papillomas ranges from 2 to 4 weeks. To elucidate whether administration of a topical antiviral drug may shorten the recovery time in ESP patients follow surgery, we tested the effect of rhIFN-α2b. We found that administration of rhIFN-α2 following surgical removal of ESP significantly increased the rate of resolution of postoperative reactive phenomena, suggesting better healing and repair processes in the nasal mucosa. A similar resorptive effect of rhIFN-α2b was demonstrated in patients with hypertrophic scars and keloids.^15^ This could be explained by the fact that IFN-α (Interferon-alpha) enhances the human response to Th1 (Type 1 T-helper). It helps restore Th1/Th2 (Type 2 T-helper) balance in Th2-predominant diseases by downregulating Th2 cytokines such as IL-4, IL-5 and IL-13. rhIFN-α2b increases the expression of anti-inflammatory IL-10, inhibits IFN-γ (Interferon‐gamma), TNF-α, and enhances the regulatory activity of T-lymphocytes and Natural Killer cells (NK).^12^, ^13^, ^14^, ^16^, ^17^ In addition to the defect of the T-helper link, papillomavirus infection is accompanied by inhibition of interferogenesis and, ultimately, against the background of immune disorders, HPV initiates the development of secondary immunodeficiency, which in turn leads to a slowdown in regeneration processes and maintenance of inflammation.^16^, ^17^, ^18^ The effect of the drug containing interferon contributed to the acceleration of the resolution of reactive phenomena that arose under the influence of HPV by compensating and eliminating the phenomena of immunodeficiency. A number of studies have also shown that rhIFN- α2b can bind to interferon receptors on the surface of target cells, promote the expression of antiviral proteins, inhibit HPV replication, reduce the expression level of viral proteins and restore the focus of infection to a normal state.^19^ A decrease in HPV concentration due to the antiviral effect of rhIFN- α b contributes to a more active recovery process in the postoperative area.

Sinonasal papillomas have a tendency for recurrence, reaching 60% within 5 years of initial detection, and malignant transformation.^12^ Therefore, as a next step, we sought to determine whether the supplementary treatment with rhIFN-α2b reduces the rate of ESP recurrence. We found that a 6-month recurrence rate of ESP was significantly lower in patients who received the supplementary rhIFN-α2b treatment, compared to control. Meanwhile, the lack of significant differences between Groups A and B in the recurrence frequency during the first 3 months of observation is likely associated with the effectiveness of the surgical treatment performed in both groups and the short duration of observation, during which the virus did not have sufficient time to cause a recurrence. Remarkably, administration of rhIFN-α2b but not category of HPV oncogenic risk was associated with the recurrence rate. A similar effect of interferon treatment was demonstrated in patients with HPV-associated papillomas of the lacrimal sac, urogenital tract, and other areas of the body.^13^, ^14^, ^15^ Reduction in the ESP recurrence rate observed in our rhIFN-α2b-treated group could be explained by antitumor activity of the drug. First and foremost, IFN-α2b can directly act on malignant cells and induce cell cycle arrest, apoptosis, and angiogenesis inhibition, having a strong impact on tumour initiation and progression. Secondly, IFN-α2b can stimulate the indirect effects including immunomodulatory effects on the tumour microenvironment, including enhanced proliferation, maturation and antigen presentation of immune cells such as dendritic cells, macrophages, and NK cells, which strengthens the innate and adaptive immunity to causative agents and malignancy. rhIFN-α2b promotes the expression of antiviral proteins, inhibits HPV replication, reduces the expression level of viral proteins and restores the focus of infection.^19^ Reduction of HPV concentration due to the antiviral effect of rhIFN-α2b helps to reduce the risk of malignant transformation.^20^, ^21^

To the best of our knowledge, this is the only study demonstrating that topical administration of rhIFN-α2b in the postoperative period shortens the recovery time and reduces the rate of ESP recurrence. The strengths of our study include a large sample size, identification of HPV with high and low oncogenic risk, and a prolonged follow-up. A limitation of our study is that the tumor specimens were tested only for the presence of HPV, and therefore coinfection could not be excluded. Nonetheless, a clear difference was seen in the recovery time and ESP recurrence rate between patients who were treated with rhIFN-α2b in the postoperative period comparing with those who were not.

## Conclusion

ESP is a disease that significantly affects quality of life of the patients. We have managed to establish that the frequency of postoperative recurrences in ESP is high, especially in cases of incomplete tumor removal, and is associated with HPV. In the present study we demonstrated that topical application of rhIFN-α2b following surgical excision of ESP alleviates the postoperative reactive phenomena, shortens the recovery time, and decreases the 6-month tumour recurrence rate.

## Authors’ contributions

Popova Inga: Conceptualization; Project administration; writing (original draft preparation); funding acquisition.

Tregub Pavel: Validation; Project administration.

Degtyarevskaya Tatiana: Conceptualization.

Starostina Svetlana: Methodology; Validation.

Shadyev Timur: Methodology; Visualization.

Apollonova Irina: Validation.

Boyko Andrey: Methodology; Visualization.

Petrovskii Vladimir: Writing (original draft preparation).

Kozlova Anastasia: Writing (review and editing).

Ibrahimli Irada: Writing (review and editing).

All authors have read and agreed to the published version of the manuscript. All the authors have made a significant contribution to the preparation of the scientific work. A large number of authors are associated with a significant volume of the patient group and the interdisciplinary nature of the study.

## Funding

This study did not receive any specific grant from funding agencies in the public, commercial, or non-profit sectors.

## Conflicts of interest

The authors declare no conflicts of interest.
